# Unpacking the impact of integrating the neglected tropical disease supply chain into the national supply chain system: illustrative evidence from Liberia

**DOI:** 10.1017/S0031182023000896

**Published:** 2023-09

**Authors:** Karsor K. Kollie, Jack Jenkins, Sally Theobald, Gartee Nallo, Otis Kpadeh, Lent Jones, Darwosu Borbor, Maneesh Phillip, Anna Wickenden, Jewel Tarpeh Kollie, Emerson Rogers, Zeela Zaizay, Martyn Stewart, Laura Dean

**Affiliations:** 1 Department of Health Services, Ministry of Health-Liberia, Congo Town Back Road, Monrovia, Liberia; 2 Liverpool School of Tropical Medicine, Pembroke Place, Liverpool L3 5QA, UK; 3 University of Liberia Pacific-Institute for Research Evaluation, Monrovia, Liberia; 4 Cuttington University Graduate School, Monrovia, Liberia; 5 Effect Hope, 200-90 Allstate Pkwy, Markham, ON L3R 6H3, Canada; 6 A. M. Dogliotti School of Medicine, University of Liberia, Monrovia, Liberia; 7 Action Transforming Lives, Congo Town Backroad, Monrovia, Liberia

**Keywords:** equity, health system strengthening, integration, Liberia, medical commodities, neglected tropical disease, quality of care, supply chain

## Abstract

Effective supply chain management is a critical pillar of well-functioning health systems ensuring that medical commodities reach those in need. In Liberia, the national neglected tropical disease (NTD) programme supports health systems strengthening for case management of NTDs. Integration of NTD commodities into the national health system supply chain is central to the integrated approach; however, there is minimal evidence on enablers and barriers. Drawing on qualitative evaluation data, we illustrate that perceived benefits and strengths to integrating NTD commodities into the supply chain include leveraged storage and management capacities capitalized at lower system levels; the political will to integrate based on cost-saving and capacity strengthening potential and positive progress integrating paper-based reporting tools. Challenges remain, specifically the risk of reliance on donor funding; difficulty in accessing commodities due to bureaucratic bottlenecks; lack of inclusion of NTD commodities within electronic data tools and poor coordination leading to an inability to meet demand. Collectively, the negative consequences of ineffective integration of NTD commodities into the supply chain has a detrimental impact on health workers (including community health workers) unable to deliver the quality of care to patients. Trust between affected populations and the health system is compromised when treatments are unavailable.

## Graphical abstract

**Figure:**
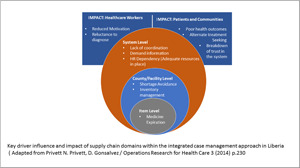

## Introduction

Effective supply chain management is a critical pillar of a well-functioning health system, ensuring that medicines and supplies reach all those who need them in time. Neglected tropical diseases (NTDs) are often described as a barometer for health equity, with many remote-rural populations thought to only access formal health services through NTD interventions (Dean, 2019). Thus, by extension, the availability and accessibility of NTD supplies and commodities can be seen as a tracer for supply chain equity. The World Health Organization (WHO), in its NTD roadmap, ‘Ending the Neglect to attain the Sustainable Development Goals: A road map for neglected tropical diseases 2021–2030’, supports this notion, stressing the need for the effective management of NTD medicines and supplies in an integrated manner in order to ensure access to all populations in need (WHO, 2021). However, despite its importance, the integration of NTD medicines and supplies within national health systems is thought to be one of the most ambitious targets for the NTD roadmap (WHO, 2021), as a consequence of the many elements and layers of complexity associated with embedding vertical programmes into an existing system. Understanding the barriers and enablers to adopt an integrated approach to NTD supply chain management is essential to ensure progress towards WHO roadmap goals and to support progress towards health equity.

Yadav et al. (2011) describe a well-designed medicine supply chain as one that ensures that ‘procurement, warehousing, and transportation are seamlessly linked to form a network that can deliver the requested medicines to health facilities and pharmacies in good time, in the correct quantities and at the lowest possible cost’ (Yadav *et al*., 2011, p. 2).

There is, however, a combination of factors that may impact a supply chain and contribute to low access to effective medicines, and other life-saving technologies, in low- and middle-income countries (LMICs). These include shortages of healthcare workers, a lack of awareness of the disease and availability of medication, severe financial constraints and inefficiency in existing systems (Yadav *et al*., 2014). Additionally, most literature on supply chains and procurement focuses on for-profit organizations/companies (Cox, 2002). For example, in Karamoja, northeast Uganda, researchers concluded that when complexity like lead time, lack of transparency and information sharing and uncertainty are reduced in the supply chain this produces higher customer service and profit maximization for private institutions and promotes integration (Jahre *et al*., 2012). Less is known about the not-for-profit supply chain integration. Supply chain integration becomes critical in supporting productivity, efficiency and cost-effectiveness, thereby allowing institutions to focus only on patient care instead of diversion of attention on other things (Yadav *et al*., 2014).

Several practices of supply chain integration are emphasized in the literature to support resource maximization (Maliyogbinda and Tijjani, 2022). For example, Maliyogbinda and Tijjani (2022) emphasize 4 best practices: (1) relationship management which is based on interconnectivity and is the basis of trust and affiliation; (2) integration of information focused on sharing information and data review from individual vertical supply chain system; (3) integration of products that is concerned with vertical supply chain pulling of resources for storage and transportation of commodities and (4) effective coordination that relies on good quality leadership for those institutions in the network.

In addition to these examples of best practices, Privett and Gonsalvez (2014) identify 10 common domains across which supply chain issues are frequently identified as shown in Table 1. These domains were identified through surveys conducted with supply chain professionals, who, they argue hold the most knowledge on the issues encountered but that are rarely captured in technical reports and papers (Privett and Gonsalvez, 2014).

**Table 1.** Ten common supply chain issue domains

Supply chain domain	Definition and importance
Supply chain coordination	Supply chain coordination is the stewardship of supply chain activities. It ensures coherence and motivation in the team, and sets standards to support supply chain effectiveness.
Inventory management	Inventory management involves tracking stock and stuck-out of commodities and supplies as well as quantification that support replenishment of commodities and supplies.
Demand information	Data generated on consumption at any level of the supply chain is regarded as demand information for supply chain. That data are useful to inform procurement and supply chain decisions.
Human resource (HR)	In supply chain HR is concerned with qualified personnel trained to effectively manage the supply chain system with the aim to provide equitable access to quality, adequate and timely commodities to those in need.
Order management	Involves planning, ordering and following up on medical commodities to support accessibility of commodities. With effective order management, there will be timely availability of commodities.
Shortage avoidance	Shortage avoidance is strategies employed by supply chain systems to mitigate the effect of shortages of medical commodities. Some of these include frequent ordering, frequent replenishment, large buffer stocks and emergency ordering.
Expiration	Expiration in the medical supply chain is when the usefulness of a commodity and supplies are rendered out of date and therefore not helpful to those in need. This usually leads to product wastage.
Warehouse management	Warehouse management issues centre around the importance of storage, organization, capacity and shared space management.
Temperature control	The temperature control domain most predominantly relates to medication and vaccines that need to be stored at a specific temperature, especially those that require a cold chain.
Shipment visibility	Tracing shipments in the supply chain after they leave the manufacturer or origin, usually becomes an issue with most prepositioning or shipment processes, becoming most challenging before reaching their final destination.

*Source*: Privett and Gonsalvez (2014).

Liberia is a West African country with an estimated population of 5 million people. It has experienced several crises in the past 3 decades including civil wars (1989–1996, 1999–2003), the Ebola virus disease epidemic (2013–2016) and the COVID-19 pandemic. The Ministry of Health of Liberia has prioritized NTDs in the revised National Health Policy and Plan (Dennis, 2006; Liberia MOH, 2010). Since 2016, the Liberian Neglected Tropical Disease Programme has been implementing a new approach to health service integration for NTDs that require case management (CM NTDs) including leprosy, Buruli ulcer (BU), yaws and the clinical manifestations of lymphatic filariasis (lymphoedema and hydrocele). Combined post-integration detection rate for all diseases is 0.116% with a rate ratio at 4.385. This meant that integrated counties would detect cases about 4 times more likely in integrated counties than in non-integrated counties. When we disaggregate the detection rate, rate of detection of lymphoedema is at 0.039%, yaws 0.0034%, 0.0197% hydrocele, BU 0.029% and leprosy 0.0242%. In terms of quality of care, 80% of BU cases adhered to treatment and 90% self-care adherence for lymphoedema cases (Kollie *et al*., 2023, pending publication). To implement the new approach, a strategic plan for the case management NTDs, which is aligned with the Liberia NTD Master Plan of 2016 and other Ministry of Health (MoH) polices and plans, was developed. This integrated approach has included CM NTD commodities in the national health system supply chain since 2017. NTD commodities in this context refer to all essential medicines and treatment supplies for yaws, BU, leprosy, lymphoedema and hydrocele. This means all essential medicines for the CM NTDs are included in the essential medicine listing of the MoH, requested through the supply chain system of the MoH, and prepositioned, packaged and managed through the central medicine stores (CMSs) of Liberia.

Case management NTDs including BU, yaws, leprosy, hydrocele and lymphoedema are managed using specific antibiotics. Clarithromycin for BU, azithromycin for yaws and leprosy treatment includes rifampicin, clofazimine, dapsone and prednisolone. Hydrocele requires surgery and other wound management approaches. Lymphoedema mainly requires home-based and symptomatic management. Other major supplies that may be required include bandages, gauze, antiseptic, adhesive plaster, hand gloves and instruments for dressing. Home-based self-care kits include anti-bacterial and fungus cream, soap, towel, etc. for lymphoedema (see Table 2).

**Table 2.** NTD management and specific medicine of choice

	NTD or condition	Medicines needed	Materials sometimes needed	Other management
1	BU	Clarithromycin	Bandages, gauze, antiseptic, adhesive plaster, hand gloves, instruments for dressing	Wound management
2	Yaws	Azithromycin	Bandages, gauze, antiseptic, adhesive plaster, hand gloves, instruments for dressing	
3	Leprosy	Rifampicin, clofazimine, dapsone prednisolone	Bandages, gauze, antiseptic, adhesive plaster, hand gloves, instruments for dressing	Wound management
4	Hydrocele		Bandages, gauze, antiseptic, adhesive plaster, hand gloves, instruments for dressing, surgical instruments like, caesars, sutures, etc.	Surgery and wound management
5	Lymphoedema		Bandages, gauze, antiseptic, adhesive plaster, hand gloves, and instruments for dressing. Home-based self-care kit including anti-bacterial, fungus cream, soap, towel, etc.	Home-based care and wound management

Prior to the integrated approach, procurement of medical products for CM NTDs was done by individual international and local partners and then donated to the NTD programme. Storage was usually done at NTD offices and medical products were frequently kept at partners' offices until ready for prepositioning by the NTD programme. Prepositioning refers to the stockpiling of equipment and supplies at or near the point of planned use (Lodree *et al*., 2012). Prepositioning of medical products to the county was done by the NTD programme based on need without the involvement of the CMS. At the county level, most of the CM medical products were managed by the county NTD focal points at their offices instead of by county supply chain pharmacists at the county depot (Republic of Liberia Ministry of Health, 2016). Anecdotally, the distribution of CM NTD commodities in this way not only places an unnecessary burden on NTD programme staff and partners but also presents challenges in the equity of resource distribution and bypasses national procurement systems. Thus, within this paper, we will look at the technical process, challenges and opportunities of integrating the CM NTD commodities within the national supply chain system to make recommendations of how best to continue this effort in Liberia and to synthesize learning for other settings.

Despite the importance of including the supply chain in the integrated model for CM NTDs in Liberia, prior to this analysis, there has been little evidence produced on the hindering and enabling factors for integrating CM NTD commodities into an existing supply chain system, or the impact of those factors on the health system, the health workforce and those affected by CM NTDs requiring treatment. It is important to understand barriers and enablers for non-profit supply chain systems within an integrated approach to support health system strengthening and health outcomes in Liberia and beyond. Hence, the aim of this study was to identify the enablers and barriers to effective integration of the CM NTD supply chain (medical commodities) into the national supply chain system in Liberia and explore the consequences of this integration of key stakeholders. We draw on Privett and Gonsalvez's domains of supply chain challenges to shape our learnings within this paper to support their dissemination to the Liberian MoH and other NTD programme managers who might be contemplating a similar approach. The results will also contribute to the debate and evidence gap on health service integration but with a focus on medicine and supplies that extends beyond the opinions of supply chain professionals.

## Methods

### Study design and site

**Figure 1.**
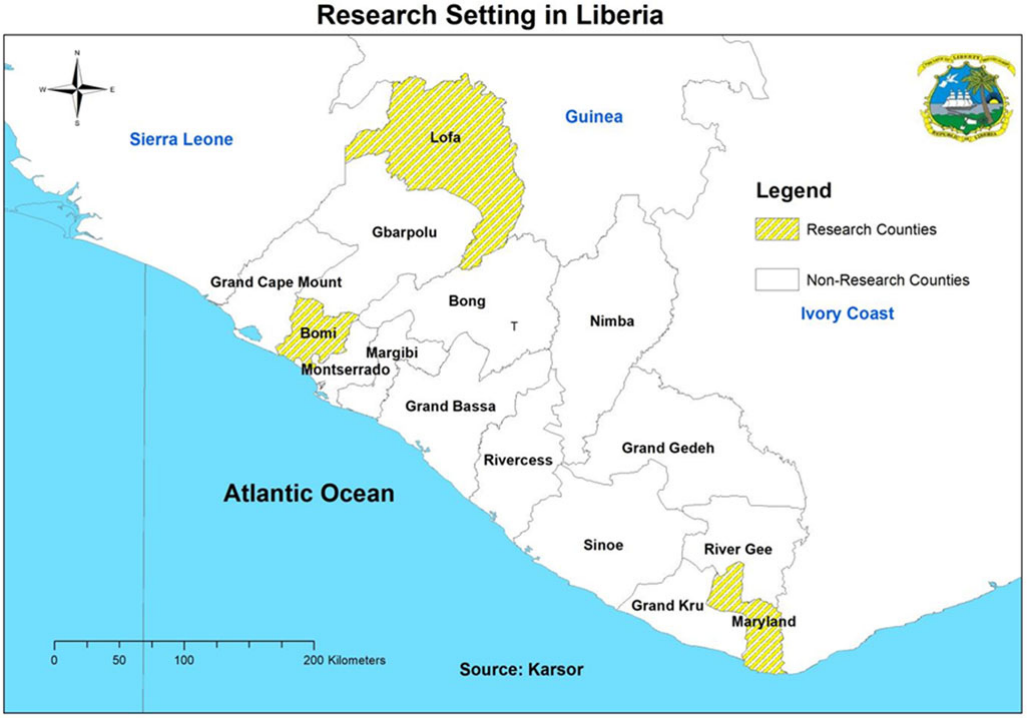
Research setting: Lofa, Maryland and Bomi counties.

To evaluate the CM NTD supply chain integration into the health system of Liberia, a qualitative evaluation method was used across 3 counties (Lofa, Maryland and Bomi) where implementation of the integrated approach is currently ongoing. Three counties were purposively selected amongst the 5 pilot integration counties (see Fig. 1) to ensure maximum variation in geographical characteristics, diverse disease burden and CM NTD co-endemicity.

Lofa county with an estimated population of 370 361 people is located in north-western Liberia and bordered by Guinea and Sierra Leone. It is endemic for BU with a prevalence at 0.2/10 000 population, lymphoedema estimated prevalence is 1.3/10 000 population, hydrocele prevalence is 0.3/10 000 population and leprosy prevalence is 0.1/10 000 population. There are no confirmed cases of yaws reported.

Lofa county has a relatively high number of CM cases. The predominantly spoken languages are Lorma, Kissi, Gbandi, Mende, Mandingo and Kpelle (MoH NTD Master plan 2023).

Maryland county has an estimated population of 181 845; it is located in south-eastern Liberia, bordered by Ivory Coast, and endemic for all 5 CM NTDs. BU prevalence is 0.21/10 000 population, yaws is prevalent at 1.3/10 000 population, lymphoedema is prevalent at 10/10 000 population (reporting the highest number of cases), hydrocele prevalence is prevalent at 6/10 000 population and leprosy prevalence is prevalent at 0.3/10 000 persons. Predominantly spoken languages are Grebo and Kru (MoH NTD Master plan 2023).

Bomi county is located in the western region of Liberia with an estimated population of 112 526: It is endemic but low for BU with a prevalence of 0.2/10 000 population, lymphoedema is prevalent at 0.8/10 000 population, hydrocele prevalence is 0.4/10 000 population and leprosy is 0.2/10 000 persons. Predominantly spoken languages are Gola and Vai (MoH NTD Master plan 2023).

A qualitative evaluation approach was used as we were concerned with understanding the process and impact of integration (phenomenon) from the perspectives of a diverse range of health system stakeholders (Stuckey, 2013*a*, 2013*b*, 2015; Cristancho *et al*., 2018). We engaged different stakeholders from all levels of the health system including the international, national, county, district and community levels. The lead author of this paper also serves as the programme manager for the national NTD control programme. Thus, interview data were complemented through the use of diary notes and observations made during programme delivery. These diary notes and observations drew on a semi-structured guide and add rigour to the interpretations of our data as they were taken by someone with such insider insights into the context (Chavez, 2008; Greene, 2014; Aburn *et al*., 2021).

### Participant selection

During 2019–2020, key informant interviews were completed to understand key stakeholders' perception of the integrated approach, their experience of the implementation, weaknesses and strengths of the approach and recommendations for improvement. The interview participants comprised international funding partners (3), national health actors (5), county health team members (3) and health facility staff (3). Three focus group discussion (FGD) sessions (24) were held with community health assistants and community health volunteers (CHAs and CHVs) to understand their experience of implementing the NTD integration approach. In-depth interviews were also facilitated with people affected by CM NTDs (6) to understand their health-seeking and treatment experiences. Across all levels of the health system, purposive sampling was used to select respondents based on their expertise or understanding of the issues and to ensure maximum variation in participant views. Key informants were largely selected based on their job role which gave insight into the factors that hinder or facilitate the CM NTD integration process. Focus group participants were selected with CHVs and CHAs because they were familiar with the topic and key issues related to community-based health integration. In-depth interview participants were selected to explore the detailed thoughts of NTD-affected persons regarding disease conditions and treatment experiences.

We interviewed 44 participants in total (24 males and 20 females). This included 14 key informants – mostly international partners, programme managers, assistant ministers, county health team members, district supervisors and clinicians that were key to the NTD implementation. The FGD was held with 24 participants in total 3 different FGD sessions. The FGD participants were mostly CHAs and CHVs who had insight into the supply chain and NTD integration. In-depth interviews were conducted with NTD-affected persons who were receiving the services (Table 3).

**Table 3.** Participant characteristics

	Key informant interview	FGD (3 sessions)	In-depth interview	Total
Positions	International partners, national programme managers, county health teams, clinicians	CHAs and CHVs	People affected by NTDs	
Male	6	16	2	24
Female	8	8	4	20
Total	14	24 participants (3 sessions)	6	44

### Data collection and management

Data collection was completed between July 2019 and May 2020 by the lead author and 3 independent research assistants. Research assistants were selected based on their previous experience in NTD research. Research assistants were trained in the context of integration by the lead author. Topic guides for each method were developed, pre-tested and refined, and finally used for data collection. Due to the positionality of the lead researcher (national NTD programme manager), research assistants supported in collating data from certain participants (e.g. national-level MoH staff and county-level supervisors) where pre-existing power dynamics may have hindered the trustworthiness of the research data.

Ethical clearance for the study was obtained from the Liberia UL-PIRE Internal Review Board (IRB) (Ref: 19-05-163) and LSTM Research Ethics Committee (Ref: 18-035). Prior to all data collection methods, research assistants contacted participants *via* phone calls or email (where possible). They also approached participants in person where possible to provide the participant with a step-by-step discussion of the participant information sheet. Following initial verbal consent, the time, date and location for the interview were set at the participants' convenience. On arrival at the interview location, the participant information sheet was re-explained, and any issues of concern for the participants were addressed. Literate participants were asked to sign the consent form and illiterate participants signed using thumb fingerprint in the presence of a witness prior to the beginning of the interview.

### Data analysis

All data were collected in English. Notes were taken and verbal responses were recorded and transcribed verbatim. Transcripts, notes and reflective diary entries were analysed together using the framework approach (Gale *et al*., 2013). Using computer-assisted software (NVivo version 11), Karsor Kollie (K. K.) led the analysis with support from the local research assistants and in discussion with the wider study team.

## Results

A platform exists for the integration of medicine and supplies for NTDs in Liberia. This includes existing structures and access to individuals with the capacity to procure and deliver medicines. However, there are both enablers of and barriers to the success of this integration for the national NTD programme, and these are discussed in the following sub-themes: parallel systems: procurement and donor reliance, funding flows and ensuring availability; medicine requisition processes: bureaucracy and reporting gaps and commodity availability: medicine transportation, protection and expiry. Collectively, these barriers and enablers have impacts on health workers' motivation and longer-term treatment outcomes for NTD-affected persons, impacting the overall effectiveness of integrated case management of NTDs within the national health system of Liberia.

### Parallel systems: procurement and donor reliance, funding flows and ensuring availability

All national and international respondents described that donor reliance and no fixed funding from the national government for the CMS to procure NTD medicines and supplies was a key factor hindering supply chain integration. National informants felt that many donors and international partners ‘they are sometimes selective on which portion of the national budget to support annually’ and supporting the provision of treatment/medicines was sometimes less favoured than other areas of the programme budget presenting a ‘major challenge for the supply chain’ and thus ‘creating a neglect among the neglected diseases in face of treatment’ (National NTD Programme key informant-04).

A few international respondents felt that limitations in medicine availability within Liberia were part of the challenge in ensuring medicine procurement within pre-existing system infrastructure, resulting in parallel procurement systems. They described a process through which frequently procure medicines externally on behalf of the NTD programme or indirectly procure by the NTD programme without the involvement of the CMS of the MoH. Such medicines were described as remaining in the custody of the NTD programme, with a limited engagement of the CMS, raising further challenges for supply chain management and medicine stewardship.

Drugs for case management are usually bought either from local vendors or ordered out of the country because some of the drugs cannot be gotten from the ministry and are not in the country. NTDs drugs are mostly not in the custody of the supply chain as expected. The drugs are usually procured and given directly to NTDs programme so, there's no assurance if NTDs programme give the drugs to the supply chain (International partner key informant).

However, at the national level, many programme leads highlighted that donor reliance and supply system limitations were what resulted in medicines remaining in NTD programme control at the national level, with most medicines re-entering the system infrastructure within counties. In their opinion, this measure was utilized to address the bureaucracy challenges described above with a view to try and enhance medicine availability.

Because the Ministry of Health can be quiet and ineffective and slow in responding because of the same logistical limitations when there are logistical limitations, the CMS cannot push drugs as fast as they should go. So sometimes we are obliged to send drugs straight from NTDs program to the county level but in the county level it enters into the supply chain system, they recaptured and recorded (Female, National Programme informant-07).

Procurement and delivery of medicines by donors and implementing partners were met with further critique, particularly when shipment visibility (i.e. pre-alert and notification of the recipient of the donated item) occurred last minute. For example, most national programme informants explained that usually the CMS or the programme is alerted when the commodities are at the port of entry or on the day of donation. They felt that this process goes against national guidelines and resulted in some commodities not being received on time, documented, transported and managed appropriately because of weakness in shipment visibility and poor coordination.

It is really frustrating that some of our partners do not follow the rules of the ministry, our donation guidelines says that the CMS and supply chain should be pre-alerted weeks before shipment arrives in country or donation to MoH, but this is not the case with some international partners, we are informed when the medicines are already at the airport for clearance. How do you expect us to clear the items from the port of entry, where do we get the logistics from to transport, even the storage capacity. In fact, sometimes you just come to the office and you are [warned] that this organization is donating medicine to the MoH today so get ready, that is not how integration works (National supply chain informant-09).

Looking to the future, the majority of international and national informants agreed that despite the challenges there are benefits to integrating NTD medicines into the supply chain system; national-level participants in particular emphasized both the cost-saving and capacity-strengthening benefits that full integration would bring.

On the other hand, with all of the challenges, integration in supply chain distribution is helpful to the NTDs programme because it reduces the management and distribution cost to facilities. With this, facilities can receive drugs from National upon their request (International partner informant-01).

### Medicine requisition processes: bureaucracy and reporting gaps

Integration of NTD commodities into the supply chain has taken place within the requisition and distribution lists, storage and prepositioning, which is a key success of integration efforts to date. However, currently, NTD commodities are not included in the electronic reporting tools. For example, due to a lack of funds and competing priority on the part of key stakeholders, NTD medicines and supplies are not yet listed on the existing stock status requisition and reporting form, and the reporting electronic platform is referred to as electronic logistics management information systems (eLMIS). This means that ongoing monitoring of NTD commodity consumption at the county and national levels is difficult, and ongoing replenishment based on consumption is impossible.

Most of the NTDs medicines are on the requisition list, but some are not on the …. Reporting electronic softcopy and others are on hard copy supply chain tools and essentials medicines list (National-level staff, supply chain key informant).

We have all the standard guidelines for supply chain including for example the NTDs SOP that we developed through the support of ASCEND, but the problem is implementation of all these documents (National level staff, supply chain key informant).

National and county level participants expressed those high levels of bureaucracy result in lengthy medicine requisition processes, request delays and resultant inaccessibility of essential medicines and supplies. They also added that pre-determined quarterly distribution timelines can result in protracted periods without medicines, particularly where 1 request cycle has been missed and compounded by further lengthy bureaucracy for emergency request.

CMS supplies (pre-positions) on a quarterly basis, so if a county does not make a quarterly requisition on time and then the supply is not done, that particular county will not have access to case management NTD drugs, [and] that means patients will go out of medications for a good while. Another option is we can put in for an emergency request but that one, the procedure is long and all logistics cost to deliver the items to the counties will be undertaken by the NTDs programme, not the CMS (National level staff, NTD programme key informant).

For medicines only available through request from the WHO [e.g. multi-drug therapy (MDT) for leprosy] lengthy bureaucracy was seen as a challenge in receiving medicines in a timely way.

One of the problems the programme is facing about NTDs medicines is that some are only [accessed] through the WHO, you cannot even get it to buy. To get medicines through WHO is helpful but can delay a lot (National-level staff, NTD programme key informant).

### Commodity availability: medicine transportation, protection and expiry

Due to the cost and scarcity of necessary medicines and commodities within Liberia, some national-level respondents described making a choice not to preposition NTD medicines at health facilities. They explained that this was primarily to avoid medicines for NTDs being used by other health programmes and to avoid expiration. These informants stressed the importance of only releasing medicines from national or county depots (when available) upon confirmation of a case management in an NTD case. Consequently, this means that medicines are not always available when cases are initially identified and are further delayed based on lengthy requisition processes articulated above.

The issues of NTDs medicine especially specific antibiotics, the medicines are very expensive and sometimes we cannot see the medicine in-country to buy. So sometimes we do not want the health facility clinician or the county health team to use the small stock of medicines for something else or if we carry early maybe if they do not find cases (CM NTD) in that health facility the medicine will still [be] there and get outdated. Or they will use it for something else (Illnesses other than CM NTDs) then when we find cases the medicine is not available, so it is better to keep it until we find cases first (National programme staff informant-05).

However, many informants also described finding solutions to the medicine availability dilemma as essential, describing that adjusting and taking small risks by prepositioning NTD medicines at each health facility might support the integration process. They also articulated that such prepositioning would need to be accompanied by appropriate steps to ensure medicine protection and stewardship.

I think it might be wise to preposition some quantity of essential NTDs medicine at various health facilities for easy access and availability when needed. The value of having medicine prepositioned is [outweighed by] not having any when you need it (National NTD programme staff informant).

Most national-level participants described that existing infrastructure within the CMS meant that storage and management of medicines within the routine supply system is possible for NTD medicines. These perceptions were validated by diary notes made by the lead author during field visits and programme supervision; however, diary notes also emphasized challenges in the storage capacity of the current supply system. For example, frequent electricity outages and occasional breakdown of trucks used to transport medicines and supplies to county depots may lead to medicine wastage and delay transport. Further, an absence of logistical support and funding limited the movement of NTD medicines and commodities through pre-existing systems infrastructure.

Most [of the] time, drugs [used] to treat cases are not available but sometimes the drugs are available but there are no means to transport [them] to the facility, and most of the areas here are hard-to-reach areas, very difficult. You have rivers to cross, you have deep valleys to cross, sometimes it requires stopping at one end and the people come for the drugs on the order side, very difficult circumstances (Key informant Bomi county-02).

Some national and international key informants described the importance of CMS taking the responsibility of managing NTDs medicines and supplies as this reduces the burden on the NTDs programme to transport and properly manage medical commodities for use by the NTDs programme (Diary note, 19 August 2019, Health Services Monthly Meeting).

Most medicines and medical supplies for NTDs are managed by the central medicine store of Liberia instead of management by the programme itself. This has [relieved] the programme of the burden of proper storage and transportation of medicine and medical supplies to health facilities (National-level KII-10).

Some informants felt that inability to meet the demand for medicines compromised the overall quality of the case management programme and was creating a significant treatment gap. Fears were also articulated in relation to treatment interruption and the risk of contributing towards antibiotic resistance.

We identify people with cases, but we don't have enough drugs to cater to them. So there is this gap, …. between us, the Ministry of Health, and the ultimate beneficiaries; because when you identify the people you create expectation, if you don't have the supply now to cater to that expectation, that is ethically wrong. This makes the patients doubt the program ability and the patients have no option but to result to traditional medicine which might worsen the patient's condition (International partner key informant-05).

The problem of antibiotic resistance is one thing for the programme to take serious in the midst of medicine stock-out from the health facilities. We cannot be giving a half dose of medicine to patients and when they come refill and then we say no medicines (International partner key informant-07).

### Weak supply chain impacts health workers: staff attrition-motivation and breakdown in trust

Most key informants at the clinic and county levels agreed that challenges in ensuring adequate NTD medicines and supplies (as a result of factors described above) led to frequent stock-outs at the health facilities. Most informants described that stock-outs had led to many health workers becoming demotivated, frustrated and with a desire to leave when they cannot treat cases before them at the facility. A female health facility clinician had this to say:

As it is, it is better for me to leave this clinic if I cannot even get an injection needle or a pair of gloves non [sic] to talk about medicines to treat the sick. It is painful and demotivating to stand by a patient till they die from curable conditions only because you do not have the medicine and medical supplies to treat them (Lofa county health facility informant-09).

During FGDs, CHAs and CHVs across all counties (Lofa, Maryland and Bomi) also expressed concerns about medicine stock-outs and limited availability within health facilities. They described how this compromises the quality-of-service delivery and ultimately leads to a breakdown in trust between them and their communities when they refer patients who cannot be treated. Collectively, this leads to diminished morale and CHA/CHV attrition.

We can appear as lying to the patients, sometimes we ride commercial motorbikes, and you know how motorcyclists are very expensive, that patients will fight by all means and get to the health facility but when they get there, they (clinicians) will tell them (patients) we don't have the equipment to clean and dress the ulcer. Sometimes, they (Patients) do not have transportation and so they end up going back to the village and find some kind of traditional herbalist. That can discourage them; that will also break our system down, they (community members) will think we are lying to them. So, sometimes these are some of the bad things we [have] experienced in the field (Female, Bomi county FGD-07).

### Impact on affected persons: trust issues

Most affected persons highlighted that the absence of NTD medicines and commodities in the health centre led to them seeking alternative treatment away from the formal health system, particularly when they were sent to buy medicines that they could not afford. For some participants, this left them ‘feeling bad’ which was further compounded when they felt the inability of staff to dress their ‘sore’ was also underpinned by stigma. Delayed treatment seeking, interrupted treatment provision and alternative treatment seeking has the potential to lead to the mismanagement of NTDs and ultimately hindering patient treatment outcomes.

Yes, sometimes when I go there, they can tell me say the medicine is not here you have to buy it in the drug store, they can only write it down on the paper for me to go buy it. So if we cannot get the medicine from the hospital and we na (not) get money to buy it, I end up going to the medicine man (informal health system) (Lofa county, male IDI-02).

This time, I don't go to that clinic again, I can feel bad when I go there, the doctors don't want even to touch my sore (wound). I am cleaning (dressing) my own sore now because they say no materials for dressing sore, I will go to a different place but not that place (clinic) (Lofa county, female IDI-04).

Additionally, some conditions are treated for months, and should there be a stock-out at the health facility where treatment is access free of direct out-of-pocket cost, the patients will be issued prescriptions and will cost higher as most of the drugs are expensive and sometimes not available, the patient might not afford to pay (International partner key informant-03).

## Discussion

The evaluation of the integration of CM NTD medicines and supplies into the national supply chain in Liberia demonstrates the importance of health service integration as part of an overall NTD integrated approach. Our findings illustrate the potential that supply chain integration provides for system strengthening if implemented effectively within Liberia and beyond. We have presented the views of health system actors including international partners, national health actors, county health team members, clinicians and people affected by CM NTDs to explore the current strengths and weaknesses of the process of supply chain system integration in the Liberian context. Perceived benefits and strengths to integrating NTD commodities into the national supply chain include improved storage and management capacities within the routine supply system, which are capitalized at lower system (county) levels; the political will to integrate based on cost-saving and capacity strengthening potential and positive progress in integrating paper-based reporting tools. However, challenges remain, specifically the risk of the reliance on donor funding for the national health supply chain; difficulty in accessing commodities due to bureaucratic bottlenecks; lack of inclusion of CM NTD commodities within electronic data reporting tools and poor coordination leading to an inability to meet medicine and commodity demand. This study also provides a unique contribution to the literature as we have considered and exposed the negative consequences of supply chain weaknesses on health workers including community health workers' motivation and the trust and engagement between the affected populations and the health system. Understanding and addressing the barriers identified within this study is critical to ensuring trust and confidence in the integrated CM NTD programme is maintained amongst community health workers and NTD-affected communities, ultimately shaping the success or failure of NTD programme integration.

**Figure 2.**
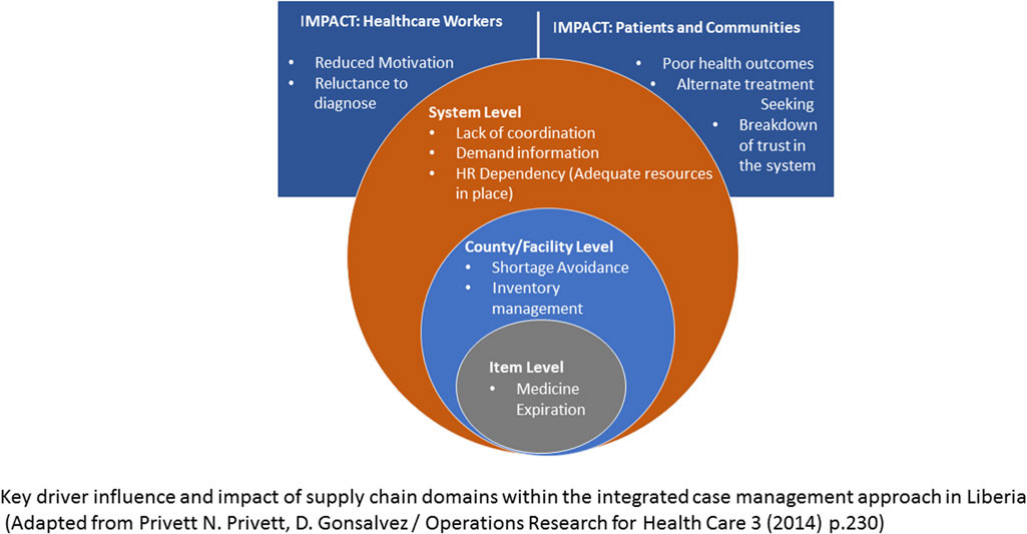
Adapted supply chain domains.

In our study, as shown in Fig. 2, we have identified supply chain issues across several of Privett and Gonsalvez's (2014) top 10 domains (Table 1). We extend the framework presented by Privett and Gonsalvez (2014) to emphasize how limitations in the supply chain have a major impact on health workers (including CHAs and CHVs) and CM NTD-affected populations. Throughout this discussion, our findings are presented in relation to Privett and Gonsalvez's (2014) supply chain domains of interest to this study.

### National-level domains

Key intersecting national-level domains shaping supply chains in Liberia include coordination, inventory management, human resource dependency, order management, shortage avoidance, expiration and shipment visibility. Our findings on *supply chain coordination* reflect literature from elsewhere, in that systems of delivery of medicines and supplies are siloed, fragmented and ultimately uncoordinated (Privett and Gonsalvez, 2014). Coordination of global health supply chains is extremely complex and many of the problems observed in global health supply chains are due to the complex interactions between multiple stakeholders that are commonly shaped by ‘intertwined and convoluted social, economic and political interests’ with conflicting objectives (Kraiselburd and Yadav, 2013), as illuminated through our case study of Liberia.

Until recently (2018), the Liberian supply chain did not include NTD medicines, reflecting findings in the generalized literature that in-country supply chains are often separate but parallel, with further division by product types and funding entities, and with decisions on where and when to send an order depending on the funder, product or project (Privett and Gonsalvez, 2014). Such complex systems make it difficult to manage and distribute medical products, and as emphasized in our data confounded by additional internal and external parallel procurement processes being implemented on behalf of the NTD programme by international partners without proper coordination with the CMS. Broader socio-political structures shape these processes, largely due to financial reliance on donors, presenting complex power relations which continue to shape supply chain decision-making in Liberia and lead to poor coordination.

Liberia faces similar issues identified elsewhere with regards to ineffective *inventory management*, including inventory inaccuracies, quantification, uninformed push systems, inventory allocation and product availability management (Privett and Gonsalvez, 2014). Inventory inaccuracy and quantification emerged as particularly challenging issues. In addition, NTD medicines and supplies are not yet listed on the existing stock status requisition and reporting form or the reporting electronic platform (eLMIS), making commodity monitoring and accurate quantification challenging and effective commodity replenishment impossible. In their study spanning 4000 health facilities across 5 developing countries, Karimi *et al*. (2021) find that the likelihood of stockouts increases with an expansion in the range of health commodities offered through the public health supply chain, with a more severe detrimental impact on resource-constrained rural facilities relative to their urban counterparts. The listing of NTD medicines and supplies on Liberia's eLMIS may be a key step towards improved inventory management and reduced risk of stockouts across both urban and rural health facilities in Liberia; however, this integration must be monitored with caution to ensure that it does not exacerbate existing challenges and ongoing stockouts due to over-diversification of the supply chain.

From a *human resource* (HR) perspective, a lack of trained supply chain staff at warehouses is a common bottleneck in many systems, leaving medical personnel responsible for making supply chain calculations and decisions, and necessitating the few qualified supply chain staff to bear heavy workloads (Privett and Gonsalvez, 2014). HR capacity is put under strain by high workloads, lack of training, deficient facilities, poor working conditions and inadequate pay, all of which combine to negatively impact employee morale and turnover. Kasonde and Steele (2017) highlight key areas that should inform the pillars of an HR capacity development plan within supply and logistics systems, including HR *planning* (policies, procedures, recruitment), *management* (job descriptions, working conditions, supportive supervision, performance monitoring) and *development* (training, certification, career paths) of the workforce [which] must all be addressed in a comprehensive and systematic manner in order to really make an impact. Within our study, rather than considering HR supply chain capacity, informants focused much more on how supply chain weaknesses impacted the general health workforce. For example, it was evidenced that stock-outs, etc. had a significant impact on health worker retention and motivation. Nevertheless, informants also felt that the key strengths of the national supply chain were the pre-existing infrastructure and the availability of trained pharmacists in supply chain management. Continued development of HR workforce capacity would therefore maintain and further enhance existing strengths of the national supply chain.

We found key *order management* challenges that affected the CM NTD programme and the national supply chain integration. Despite certain CM NTD medicines (e.g. MDT for leprosy) being supplied through the WHO, a lengthy bureaucratic process can create a ‘lead time’ challenge, further exacerbated by poor planning. This is not uncommon, for example, Jahre *et al*. (2012) found Uganda's drug supply chain to be characterized by inconsistent planning, forecasting, ordering and inventory management largely due to unclear division of responsibility between actors which, when combined with lacking exchange of information, resulted in goods flow challenges. Key recommendations for reducing the complexity of such a system included placing less supply chain responsibility on the health centres and instead developing stronger district supply chain hubs which, by moving order points ‘upstream’, allow for more frequent ordering thus reducing demand uncertainty. These lessons around the importance of reducing complexity and ensuring clarity around the division of responsibility between actors and health system levels may also help Liberia to formulate practicable strategies for overcoming some of the key order management challenges found in our research. For example, empowering districts to support order management can afford health centre staff more time to treat patients and focus on reporting correct information about stock levels (Jahre *et al*., 2012).

Despite substantial financial aid from international donors for the procurement of health products, many African countries often face stock-outs of essential drugs (Gallien *et al*., 2016); our data show that Liberia is no exception to this pattern. As Karimi *et al*. (2021) explain, there is little by way of systematic and rigorous empirical research that sheds light on the factors that drive commodity stock-outs in LMICs. Emergency ordering to address *shortage avoidance* comes at a premium (Privett and Gonsalvez, 2014). We found that additional costs for the fuel and personnel to transport commodities from the national level to the counties are experienced as disbursement falls outside of quarterly medicines requisition and distribution. In addition, emergency orders are filled from local markets, and this poses a high risk of ordering low-quality products and disrupting or interrupting the flow of other orders in the system. Additionally, we found that the Liberia CM NTD approach was to withhold CM NTD commodities at CMSs, avoiding the preposition to limit *product wastage* through misuse and expiration where limited or no CM NTD cases may be identified. Some of the concerns regarding the prepositioning of NTD commodities that arose during our research have also been raised elsewhere in regard to the prepositioning of relief supplies for the purpose of disaster preparedness (Duran *et al*., 2011; Kunz *et al*., 2014). However, our evidence shows that the benefits of prepositioning commodities outweigh the potential negatives of such a strategy and should be continued as an essential component of CM NTD supply chain integration.

Yadav (2014) describes the lack of information capture and information sharing as one of the main sources of underperformance in public sector supply chains. Similarly, Kasparis *et al*. (2021) studied the impact of information sharing through NTD delivery and performance for countries making their data publicly accessible on the performance of the NTD Mass Drug Administration (MDA) drug donation supply chain through the WHO. They found that information sharing is positively associated with the timeliness of several key stages in the NTD supply chain. Additionally, information sharing and coordination appear to be more impactful when information is released publicly and especially by country programme staff instead of international partners (Kasparis *et al.*, 2021).

If they are to perform well while maintaining cost-effectiveness, supply chains need information about stock, consumption, shipments and other such variables almost in real time (Kasparis *et al.*, 2021). Privett and Gonsalvez (2014) outline how communication worsens and *shipment visibility* deteriorates as products move further down in-country supply chains, leaving those at the lower level of the health system with little information as to when supplies will arrive. Similarly, we found that there was poor coordination with pre-alert and notification of recipients of donated items, reinforcing the dependencies that exist between the different domains of the supply chain. Yadav (2014) suggests that given the typically weak information connectivity and weak stock requisitioning capacity of rural clinics, it may be effective to couple information collection and product distribution, giving the example of the delivery team topping up system used in Zimbabwe, in which district staff collect stock information and resupply health centres in the same visit. Similar systems have been used for vaccines in Mozambique and for reproductive health products in Senegal, demonstrating similar improvements in supply chain effectiveness (Yadav, 2014). Pharmaceutical donors providing medicines for NTD MDA initially shipped to the beneficiaries' countries' port of entry, thereby leaving country programme managers responsible for customs clearance and to also delivery to CMSs leading to visibility challenges.

This was a solution through strengthening of information sharing and better coordination. The delivery was then shifted to Deutsche Post DHL Group (DHL) which created a dedicated control tower within the DHL global humanitarian logistics competence allowing the pharmaceutical donors to be involved and ensuring delivery to CMSs that led to cost saving thereby improving accountability, efficiency and transparency (Souza *et al*., 2020). Combining information collection and product distribution in this way may be of interest to stakeholders in Liberia as they seek to improve shipment visibility within the supply chain for NTD commodities.

### National/county/facility level

We did not specifically ask questions about *warehouse management*, but some dissatisfaction about the supply chain expressed by respondents is likely to be related in part to warehouse management. For example, in Liberia, NTD medicines have, at times, been stored in several locations including non-governmental organization offices partly because of the limited storage capacity following a fire at one of the main storage warehouses in 2016 (MoH, 2017; National Drug Service, 2017) (unpublished report). NTDs are not altogether integrated into the CMS platform, which manages the supplies in the warehouse. Specific medicines required for CM NTDs do not require a cold chain and there were no reports from participants related to the quality of medicines, so we have not identified this as being a critical area of concern in the context of the integrated CM NTD programme.

### Health facilities

Privett and Gonsalvez (2014) state that many health facilities see real-user demand daily, but in systems that use paper-based logs, this *demand information* is most often not shared with other levels of the supply chain, leaving most stages of the supply chain without up-to-date information. Additionally, Karimi *et al*. (2021) note that paper-based LMIS results in significant lead times for the transmission of information from health facilities to upstream supply locations, and once received reports must be manually processed, further inflating order processing times. eLMIS can therefore address these shortcomings by allowing data to be transmitted quickly and predictably by electronic means. However, in the supply chain system of Liberia, specifically in relation to CM NTD resources and commodities, our findings indicated the absence of paper-based or a lack of ability for capturing the consumption of NTD commodities. This led to the unavailability of medicines and supplies based on insufficient information to inform decision-making for quantification and procurement. As part of integration efforts, CM NTD commodities have recently been integrated within requisition and distribution lists, storage and prepositioning. However, we found that NTD commodities are not included in electronic reporting tools such as the existing stock status and reporting form or on the eLMIS. Daily reporting and eventual transition to eLMIS could potentially be transformative in overcoming some of the challenges voiced by respondents during our study.

#### An interconnected system

As shown in Fig. 2, our findings demonstrate synergies between the domains, when 1 domain is affected, other domains are compromised, hence working towards the effectiveness of each of the 10 domains will bring about efficiency in the supply chain. Table 4 distils key recommendations for supporting the integration of the vertical NTD supply chain into the national supply chain system of Liberia.

**Table 4.** Recommendations to support the integration of the NTD medicines and supplies into the national supply chain system of Liberia

National level	
Coordination	The Liberian MoH should take ownership of the supply chain system and ensure all partners pay due regard to guidelines and standard operating procedures to ensure effectiveness and best use of scarce resources.
Inventory management	Full integration of CM NTD medical commodities into all aspects of the national supply chain system, including the addition of NTDs in the eLMIS is required together with periodic review and recording of stock at all levels and routine sharing of stock reports. Including commodities on all supply chain tools necessary for effective integration may also enhance inventory management and ownership by the government.
HR	Instituting strategic and responsive mechanisms to avert stock-out in the supply chain system, will also mitigate against health worker turnover and de-motivation.
Order management	In the midst of national-level supply chain challenges, we would recommend multiple sources of some of these CM NTD medicines like for example the MDT for leprosy and an established mechanism to monitor the sources so that the quality of these medicines are not compromised. This would probably reduce bureaucratic processes at supplier level thereby reducing ‘lead time challenge’ and increasing access to CM NTD medicines.
Shortage avoidance	Avoiding ‘emergency ordering’ as a shortage avoidance strategy during the integration of CM NTDs into the larger health system as can have unintended consequences such as the NTD programme to single handily shouldering the responsibility of transportation of the emergency medical commodities to the counties and onwards to the health facilities.
Expiration	Advocacy with the WHO, national government and supply chain policymakers for procurement of adequate CM NTD medical products and prepositioning ahead of the diagnosis of cases and subsequent treatment is required.
Shipment visibility	Countries need to take ownership of their national supply chain system and ensure that international and local partners align and fully adhere to national policies and guidelines providing direction for supply chain activities to mitigate challenges introduced by ineffective shipment visibility.
National/county/facility level	
Warehouse management	Including NTDs into the CMS platform and ensuring sufficient, storage, quantity of medications and medical supplies are available and stored appropriately. This would contribute to supply chain efficiency and reduce need for prepositioning.
Temperature control	Not a critical area of concern in the context of the integrated CM NTD programme.
Health facilities	
Demand information	Ensuring the capturing and timely reporting of commodity consumption data through the eLMIS will enhance quantification and requisition.

#### Imperative of a strong supply chain as a cornerstone for integration

One of the most striking findings from this study was the significant impact that a weak supply chain had on key stakeholders and the health system in the integrated approach to NTD case management, specifically health workers, community members and people affected by NTDs. Regarding health workers, the supply chain challenges led to staff attrition and a breakdown of trust. This is critical because the training that often attracts significant attention and investment was being undermined by the supply chain. This led to a reluctance amongst health workers to diagnose CM conditions as they could not guarantee that they could provide the treatment that was needed. This was further exacerbated by the experience of patients themselves, who, following the community health worker suspecting a CM NTD condition and referring them to health centres, were unable to access the medication they needed. The impact of this experience on the overall trust of communities in the health system is critical to understand and address, and its impact is likely to go significantly beyond CM NTDs and instead impact community trust and practice in the health system more broadly.

## Strength, limitation and conclusion

The study's major strength was the design to obtain holistic opinions and views of stakeholders across the health system, including international partners, national-level health actors, county health team supervisors, health facility clinicians, CHAs, CHAs and people affected by case management NTDs. One limitation was that the study did not explicitly assess the existing infrastructure of the supply chain but rather the operationalization of the integration within the infrastructure. Additionally, the existing supply chain infrastructure did not emerge through our interactions which probably limited us on other components of the 10 domains including temperature measurement and warehouse management. These areas may require further consideration in future studies to ensure a holistic understanding of the supply chain. Additionally, further research might be necessary to look at disease-specific challenges impacting the integrating approach. Integration of the medicines and supplies for the case management of NTDs within the national supply chain is critical to the effective integration of CM NTDs into the health system. Additionally, strengthening the supply chain system warrants specific attention and investment to ensure that it does not undermine the entire CM NTD integration effort. Most critical is the need to ensure a supply chain management system that provides commodities in a timely way to those that need them to ensure positive treatment outcomes and the maintenance of trust between community members, patients, health workers and the broader health system.

## Data Availability

Data will be made accessible on request.
